# A First Assessment of SARS-CoV-2 Circulation in Bats of Central–Southern Italy

**DOI:** 10.3390/pathogens11070742

**Published:** 2022-06-29

**Authors:** Hiba Dakroub, Danilo Russo, Luca Cistrone, Francesco Serra, Giovanna Fusco, Esterina De Carlo, Maria Grazia Amoroso

**Affiliations:** 1 Wildlife Research Unit, Dipartimento di Agraria, Università degli Studi di Napoli Federico II, Via Università, 100, 80055 Portici, Italy; hiba.dakroub@unina.it (H.D.); danrusso@unina.it (D.R.); 2 Department of Animal Health, Istituto Zooprofilattico Sperimentale del Mezzogiorno, Via Salute,2, 80055 Portici, Italy; francesco.serra@izsmportici.it (F.S.); giovanna.fusco@izsmportici.it (G.F.); esterina.decarlo@izsmportici.it (E.D.C.); 3 Forestry and Conservation, Via Botticelli, 14, 03043 Cassino, Italy; luca.cistrone@gmail.com

**Keywords:** bats, coronaviruses, SARS-CoV-2, spillback, zoonotic viruses

## Abstract

One serious concern associated with the SARS-CoV-2 pandemic is that the virus might spill back from humans to wildlife, which would render some animal species reservoirs of the human virus. We assessed the potential circulation of SARS-CoV-2 caused by reverse infection from humans to bats, by performing bat surveillance from different sites in Central–Southern Italy. We restricted our survey to sampling techniques that are minimally invasive and can therefore be broadly applied by non-medical operators such as bat workers. We collected 240 droppings or saliva from 129 bats and tested them using specific and general primers for SARS-CoV-2 and coronaviruses, respectively. All samples (127 nasal swabs and 113 faecal droppings) were negative for SARS-CoV-2, and these results were confirmed by testing the samples with the Droplet Digital PCR. Additionally, pancoronavirus end-point RT-PCR was performed, and no sample showed specific bands. This outcome is a first step towards a better understanding of the reverse transmission of this virus to bats. Although the occurrence of a reverse zoonotic pattern can only be fully established by serological testing, the latter might represent an in-depth follow-up to a broad-scale preliminary assessment performed with our approach. We encourage the systematic surveillance of bats to help prevent reverse zoonotic episodes that would jeopardize human health, as well as biodiversity conservation and management.

## 1. Introduction

In late December 2019, cases of pneumonia with unknown aetiology were reported in the city of Wuhan, China [1]. The causative agent, identified as Severe Acute Respiratory Syndrome Coronavirus 2 (SARS-CoV-2), is closely related to SARS-CoV, which was responsible for the 2003 SARS outbreak [2]. SARS-CoV-2 caused a sizable epidemic of coronavirus disease 19 (COVID-19) in China, whichspread globally and was declared a pandemic in March 2020 [3]. At the time of writing, there are 476,374,234 confirmed cases of COVID-19, including 6,108,976 deaths, according to the World Health Organization (WHO) (https://COVID19.who.int) (accessed on 26 March 2022).

SARS-CoV-2 belongs to the subfamily Coronavirinae, family Coronaviridae, order Nidovirales [4]. The evolutionary origin of SARS-CoV-2 is still unknown, but it is most probably zoonotic since the virus is approximately 79% similar to SARS-CoV, which is found in wildlife [5]. Moreover, SARS-CoV-2 shares a high level of genetic similarity (96.3%) with the bat coronavirus RaTG13 [6], which was isolated from bats in Yunnan in 2013; however, none of the known bat SARS-CoVs are thought to be the immediate ancestor of SARS-CoV-2 [7].

The possible causes of the epidemiological origin of SARS-CoV-2 are unclear but the spillover is suspected to be linked to the consumption of wildlife. Based on epidemiological data, the outbreak originated in the city of Wuhan in Hubei Province of central China [8]. Two of the three earliest documented COVID-19 cases were directly linked to a market selling wild animals, as were 28% of all cases reported in December 2019 [5].

While the epidemiological route that led to the spillover is far from being fully understood, one serious concern associated with this pandemic is that the virus might spill back from humans to wildlife, which could render some animal species reservoirs of the human virus. This would be especially problematic if the species in question are threatened because their correct management would be jeopardized by their role as a reservoir. Reverse zoonoses pose serious risks to both human health and wildlife conservation [9], and, with reference to the COVID-19 pandemic, this topic has received a great deal of attention [10]. Recently, a joint statement by the Food and Agriculture Organization (FAO), the World Organisation for Animal Health (OIE), and the World Health Organization (WHO) highlighted that, although COVID-19 is a human pandemic, the virus may infect other animal species besides humans, and reverse zoonoses may affect the health of animal populations, promoting the evolution of new viral variants (https://www.who.int/news/item/07-03-2022-joint-statement-on-the-prioritization-of-monitoring-sars-cov-2-infection-in-wildlife-and-preventing-the-formation-of-animal-reservoirs#:~:text=Current%20knowledge%20indicates%20that%20wildlife,emergence%20of%20new%20virus%20variants, accessed on 18 April 2022). On such bases, the statement emphasizes the importance of monitoring mammalian wildlife populations for SARS-CoV-2 infection.

Although SARS-CoV-2 has never been observed in bats to date, viruses that are highly similar to SARS-CoV2 have been isolated in these mammals [11]: it is, therefore, legitimate to hypothesize that bats are more exposed than other species to being infected by humans. This is especially true since many bat species roost in urban areas, where they share buildings with humans [12], fall victim to domestic cats, which might provide an epidemiological link [13], and are handled by bat rehabilitators or researchers [14,15]. Despite experimental research failing to prove a significant risk of SARS-CoV-2 reverse zoonosis involving bats [16,17], studies on captive subjects do not fully replicate the epidemiological dynamics and environmental conditions that occur in the wild, so their outcomes warrant prudence [9]. A risk assessment exercise of SARS-CoV-2 transmission from humans to bats carried outin Australia concluded therisk to be low yet with a high degree of uncertainty [18]. To date, the number of published studies with surveillance results of SARS-CoV-2 circulation in bat populations is still small [19,20,21]. Preventing SARS-CoV-2 reverse zoonosis from humans to bats is paramount not only due to human health concerns but also because many bat species are at risk [22] and losing bats would mean losing the crucial ecosystem services they deliver in farmland, forests, and urban areas [23].

With an awareness of the importance of bat surveillance to monitor the potential risk of SARS-CoV-2 reverse infection from humans to bats, our general objective is to contribute to filling the existing knowledge gap on this topic. We test the hypothesis that bats occurring in urban areas, or their immediate surroundings, are exposed to SARS-CoV-2 infection and can therefore carry the virus. Although unambiguous, full evidence of a reverse zoonotic pattern can only be provided by a serological analysis coupled with avirus search in droppings and/or saliva.In this preliminary assessment, we adopted the method used in previous studies, such as those conducted in the UK [24]. Therefore, we conducteda virus search only to specifically explore the hypothesis that bats will shed the virus in a period of high viral circulation in the study area (from May to July 2021). The advantage of this method is that it can be applied on a large geographic scale by bat operators not trained specifically to take blood samples and routinely incorporated in all bat surveys due to its very limited invasiveness. Sampling bat blood in several countries requires special training [24] or is restricted to medical professionals only, as is the case in Italy. On the other hand, while serological analyses conclusively show the existence of contact between the virus and bat populations, blood sampling in small mammals of protected species such as bats requires specific authorization and training of operators (in some countries, this can only be carried out by medical professionals).

## 2. Results

**Figure 1:**
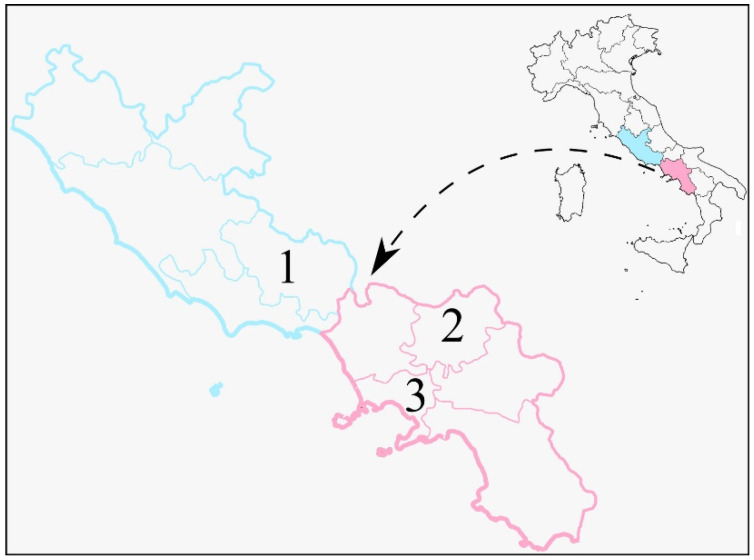
Regions of Central–Southern Italy where the bats were sampled. Sky blue colour = Lazio Region; Pink colour = Campania Region. Numbers identify the provinces where the sampling was carried out: 1—Frosinone; 2—Benevento; 3—Naples.

We sampled 129 bats from three different provinces of the Lazio and Campania Regions (Figure 1). Bats were captured under the permission of the Italian Ministry of Ecological Transition based on the positive scientific assessment made by ISPRA prot. 22990, 5 May 2021. The collected bats belonged to 10 bat species (Table 1). Specifically, we took 127 nasal swabs and 113 faecal droppings for a total of 240 sample units. All samples, from all the three provinces investigated, gave negative results for SARS-CoV-2 when analysed with the RT-PCR TaqPath^TM^ COVID-19 RT PCR kit (Thermo Fisher Scientific, Waltham, MA, USA), which is the same kit used by Istituto Zooprofilattico Sperimentale del Mezzogiorno during the pandemic for the molecular assessment of COVID-19 positivity on human swab samples. These results were confirmed by also testing the samples with the droplet digital PCR (ddPCR), using the same kit. In the provinces in which the sampling was carried out, and especially in the province of Naples, there was a high circulation of SARS-CoV-2 from March 2020 (when the COVID-19 pandemic started) until July 2021, when sampling was completed. A total of 254,049 COVID-19 human cases were reported for the province of Naples out of 2,986,745 inhabitants (accounting for 8.5% of the population), 31,663 cases in the province of Frosinone (out of 472,559 inhabitants, i.e., 6.7% of the population) and 12,731 cases in the province of Benevento (out of 266,716 inhabitants, corresponding to 4.77% of the population). Data on the number of human COVID-19 cases were taken from https://lab24.ilsole24ore.com/coronavirus (accessed on 10 May 2022), and those on the number of province inhabitants (updated 1 January 2021) were taken from www.istat.it (accessed on 10 May 2022).

To search for other coronaviruses, an end-point RT-PCR was performed with three different protocols. All samples showed no specific bands except 12 samples that yielded faint amplicons close to the expected size. These amplicons were directly sequenced, providing very low-quality sequences that made them unusable. To improve amplicon yield, we carried out the second round of PCR. Again, sequencing revealed the very low quality of the sequences, most likely due to the non-specificity of the bands.

## 3. Discussion

In our study, we found no evidence of SARS-CoV-2 presence in 10 bat species (129 individuals) sampled in Central and Southern Italy. Sampling was concentrated in summer when most of the examined bat species congregate in nurseries in buildings, a kind of behaviour that might increase the risk of reverse zoonotic transmission. The bats we sampled included both species that typically roost in buildings, quite often shared with humans, such as *P. kuhlii*, *P. pipistrellus*, *H. savii*, and *R. ferrumequinum*, and species that roost in more natural locations, especially caves (*M. schreibersii*, *M. capaccinii*, *M. emarginatus*, *R. euryale*) or tree cavities (*N. leisleri*, *M. crypticus*). While some “cave-dwelling” species may also roost in buildings (*M. emarginatus*, *R. euryale*) in the study regions considered (D. Russo, pers. obs.), in all cases, sampling took place within ca. 2 km from the closest urban site, a distance easily covered by foraging bats [25].

We also had negative results when analysing bats with generic PCR protocols, yet a band of the correct size was found for coronaviruses (CoVs) in 12 samples (corresponding to 9.8% of the total sample). However, none of the samples could be confirmed by sequencing and they were all considered negative. These data confirm those shown in a 2017 survey [26] that failed to find CoVs in 147 bats collected from three regions of Central–Southern Italy (Lazio, Campania, and Abruzzo) from 13 bat species (7 of which are featured in the present study). In [26], faint amplicons of the expected size in 10 bat samples could not be confirmed by sequencing. These data suggest that coronaviruses are absent in bat populations inthe study area that we considered, but confirmation warrants further analysis.

Two non-mutually exclusive conditions may be hypothesized to explain our negative results. First, bats might have limited or no susceptibility to a productive infection of SARS-CoV-2, as suggested by laboratory work [16,17], despite the high similarity of this virus with certain bat coronaviruses [11]. While this may be the case, it is worth highlighting that over 1400 bat species exist worldwide and are found in virtually all regions of the globe except the polar ice caps, so any generalization based on small sample sizes, restricted geographic scope, and few species warrants caution. On the other hand, transmission from humans to bats may be unlikely because, in most cases, bats have little contact with humans. Even in urbanized areas, where bats roost in spaces of buildings that can be used by people, the chances that bats will find themselves at distances close enough to be reached by droplets breathed out by a positive person are slim.

Handling bats may be a prime way to infect them, so the “spillback” risk is higher when researchers or rehabilitators, both categories professionally handling bats, are involved. In Italy, rehabilitators and researchers were promptly informed of the potential spillback risk from the very beginning of the COVID-19 outbreak, so appropriate measures were taken following the guidelines provided by the IUCN [14,15] and the Agreement on the Conservationof Populations of European Bats (EUROBATS) (https://www.eurobats.org/node/2602) (accessed on 11 May 2020). This prompt reaction may have acted as significant mitigation of the reverse transmission risk. Prescriptions made by the IUCN relied on a Minimize, Assess, Protect (MAP) strategy. It is important to minimize or avoid bat handling when it is not necessary, and in case of necessity, bat handling should not take place when operators show any symptoms of COVID-19. Moreover, practices that reduce exposure must be adopted, such as wearing masks, and gloves, and always using disinfection [14,15]. The EUROBATS panel of experts provided very similar recommendations (https://www.eurobats.org/node/2602) (accessed on 11 May 2020).

While contacts between bats and humans are rare and restricted to certain categories, the hypothesis that predation on bats by domestic cats might favour the transmission of SARS-CoV-2 from humans to bats cannot be ruled out [13]. Domestic cats are well-known predators of over 45 bat species, and due to their frequent interactions with people, they might act as SARS-CoV-2 intermediate hosts between humans and bats [13]. The recent spillover event of West Caucasian Bat Lyssavirus (WCBV) in a domestic cat from bats (supposedly, *Miniopterusschreibersii*) would provide further evidence of the role that domestic cats might play in facilitating epidemiological contact between humans and the wildlife that cats prey upon [27]. Domestic animals, such as dogs and cats [28] are susceptible to SARS-CoV-2, so we argue that the systematic surveillance of pets besides wildlife is highly important, especially where domestic animals are exposed to frequent encounters with wildlife.

We are aware that our work is preliminary, focusing on less than one-third of the bat species occurring in Italy and two out of twenty regions of the country, and that only a serological assessment of bat populations would prove the absence of infection. However, our study still provides first valuable picture, proving that bats did not shed the virus in a period of high viral circulation in humans. To our best knowledge, this is the first study dealing with the occurrence of SARS-CoV-2 in Italian bats, so we hope our contribution will encouragethe establishment of systematic surveillance of wildlife and bats, to help prevent zoonoses as well as reverse zoonotic events that would put at risk human health, as well as endanger biodiversity conservation and management. Future work will include serological analyses at specific sites and extend surveillance to other species and regions to paint a more comprehensive picture of the situation. Moreover, the virus is evolving and new variants are systematically arising. Whether further mutations will make bats more susceptible to the virus has to be established and represents another good reason to systematically continue active surveillance.

We remark that active surveillance does not replace the careful prevention of the spillback processes, which requires the strict adoption of all precautions set by the IUCN, which aim to mitigate risks for the categories of people that have frequent contact with bats.

## 4. Materials and Methods

### 4.1. Sampling

In 2021, we sampled 129 bats in different periods, i.e., May (31 bats), June (45 bats), and July (53 bats), from the Campania and Lazio regions (Central–Southern Italy) at drinking or foraging sites and near roosts with mist nets and harp traps. We collected samples from 11 bats from the province of Frosinone (Lazio), 22 from the province of Benevento (Campania), and 96 from the province of Naples (Campania). For each bat, we measured forearm length (mm) with a 0.1 precision calliper and body weight with a 0.1 g precision weigher. We established the species following Dietz et al. (2009) [29] and ascertained sex, reproductive status [30], and age class [31]. Each bat was kept in clean cotton bags for ca. 20 min before being processed, and all droppings in the bag were collected and stored in sterile vials. Saliva samples were collected with sterile swabs. All samples were immediately brought to the laboratory in a refrigerated box. Field operators tested negative for SARS-CoV-2 by molecular assay before going into the field. Moreover, the operators strictly followed the guidelines established by the IUCN [15] and took all precautions to avoid accidental transmission of SARS-CoV-2 to the bats they handled as well as any pathogen transmission from handled bats to humans.

### 4.2. Nucleic Acid Extraction

Samples underwent nucleic acids extraction by the MagMax^TM^ Viral/Pathogen II Nucleic Acid Isolation Kit (Applied Biosystems, Waltham, MA, USA) following the manufacturer’s instructions. Before extraction, oro-pharyngeal swabs were suspended in 0.6 mL of phosphate-buffered saline (PBS) and incubated at room temperature for 30 min. Droppings were suspended in 0.9 mL PBS, vigorously vortexed for 3 min, centrifuged at 13,000 rpm for 3 min, and 0.6 mL of supernatant was transferred to a clean Eppendorf tube. Then, 20 µL of protease k was added, and samples were incubated for 10 min at 70 °C. Next, 200 µL of pre-treated samples was loaded on the MagMax extraction plate, and 5 µL of internal control (included in TaqPath^TM^ COVID-19 RT-PCR kit) was added to each sample before extraction to check the quality of the extraction.

Extracted nucleic acids were eluted in 80 µL elution buffer and immediately analysed by Real-Time RT-PCR/RT-PCR or stored at −20 °C until further processing.

### 4.3. Real-Time RT-PCR for the Detection of SARS-CoV-2

The presence of SARS-CoV-2 was established by Real-Time RT-PCR using the TaqPath^TM^ COVID-19 RT-PCR kit (Thermo Fisher Scientific, Waltham, MA, USA). In the process, probes anneal to three target sequences that are specific to SARS-CoV-2. Each target is located between unique forward and reverse primers for the following genes: ORF1ab, N protein, and S protein.

The reaction was carried out following the manufacturers’ instructions. To analyse and interpret the data, we employed the Applied Biosystems™ COVID-19 Interpretive Software running into a QuantStudio™ 5 Real-Time PCR Instrument, 0.1 mL block (Applied Biosystems). Positive and negative controls were included in the kit. SARS-CoV-2 Delta variant and Omicron variant extracted from human swab samples (confirmed as positive by sequencing) were used as supplementary positive controls.

### 4.4. SARS-CoV-2 Droplet Digital PCR

To double-check for the presence of SARS-CoV2, we also carried out a ddPCR using the QX200 Droplet Digital PCR System with the One-Step RT-ddPCR Advanced Kit for Probes (Bio-Rad, Hercules, CA, USA) according to the manufacturer’s instructions. The pre-amplification mixture was prepared using 5.5 μL supermix, 2.2 μL reverse transcriptase, 1.1 μL of 300 mM DTT, and 1.25 μL TaqPath^TM^ COVID-19 RT-PCR mix, and 5.5 μL of sample template in a final volume of 22 μL. Each reaction mix was converted to droplets with the QX200 droplet generator (Bio-Rad), sealed for 5 s at 180 °C, and pre-amplified under the following cycling protocol: 50 °C for 60 min, 95 °C for 10 min, then 8 cycles of 94 °C for 30 s and 55 °C for 60 s, followed by 98 °C for 10 min, and 4 °C for 30 min. The cycled plate was then transferred and read in the FAM and HEX channels for ORF1 and N gene targets, respectively, using the QX200 reader (Bio-Rad).

The results were analysed using a droplet reader connected to a computer running the Quanta Soft Software (Bio-Rad). The feasibility of using TaqPath^TM^ COVID-19 RT-PCR mix for ddPCR was assessed by preliminary experiments on positive human samples opportunely diluted.

### 4.5. Identification of Coronaviruses by End-Point RT-PCR

To broaden the scope of our analysis, we also used three protocols to detect the general presence of coronaviruses by end-point RT-PCR. All assays were carried out with the AgPath-ID™ One-Step RT-PCR kit (Thermo Fisher Scientific, Waltham, MA, USA).

The first two protocols were carried out using the primers by Drosten et al. (2003) [32] (see Table 2) with a thermal profile indicated by Amoroso et al. (2020) [33] or with a thermal profile indicated by Drosten et al. (2003) [32] and modified as follows: 45 °C for 30 min; 95 °C for 10 min; 10 cycles of 95 °C for 10 s, 60 °C for 10 s (decreasing by 1 °C per cycle), 72 °C for 30 s; 40 cycles of 95 °C for 10 s, 56 °C for 10 s, and 72 °C for 20 s.

For both protocols, the reaction (final volume of 25 μL) contained 12.5 μL of AgPath-ID™ One-Step RT-PCR mix, 1 μL of enzyme mixture, and 1.25 μL of each of the two primers (10 μM).

In the third protocol, we used the primers mentioned by Vigen et al. (2008) [34] and the reaction (final volume 25 μL) contained 12.5 μL of AgPath-ID™ One-Step RT-PCR mix, 1 μL of enzyme mixture, and 0.3 μL of each of two primers (10 μM). The thermal profile was modified in the denaturation step with a temperature of 95 °C (instead of 94 °C) and in the extension time using 30 s instead of 1 min.

All PCR products were analysed by an automated platform Tape Station 2200 (Agilent Technologies, Santa Clara, CA, USA), using the D1000 screentape system. In all the protocols carried out, we used the following positive controls: SARS-CoV-2 Delta variant, SARS-CoV-2 Omicron variant, canine coronavirus, bovine coronavirus, and feline coronavirus. Strains were all taken from field samples, cultivated on the appropriate cell substrate and nucleic acids were extracted before they were used in the PCR assays.

### 4.6. Sequencing

Bands of interest, (251 bp or 452 bp concerning the protocol used, see Table 2) were collected using the E-Gel system (Invitrogen™, Carlsbad, CA, USA) described by Gibson et al. (2010) [35] and were either directly sequenced or underwent a second round of PCR using the same primers used in the first round.

Amplicons were sequenced as previously described [36]. The nucleotide sequence similarity searches were performed using the Basic Local Alignment Search Tool (BLAST) server (http://www.ncbi.nlm.nih.gov/genbank/index.html) (accessed on 5 September 2021).

## Figures and Tables

**Table 1:** The global conservation status of the bats examined according to the International Union for Conservation of Nature (IUCN) Red List and the location of the bat species involved in this study. The sex ratio is expressed as (males, females). VU = Vulnerable, EN = Endangered, NT = Near Threatened, NE = Not Evaluated, LC = Least Concern. Sites were categorised into urban and peri-urban.

Species	IUCN Red List Classification	Frosinone (Urban)	Benevento (Peri-Urban)	Naples (Peri-Urban)
***Miniopterus schreibersii***	VU	(0,0)	(4,2)	(0,0)
***Myotis capaccinii***	EN	(0,0)	(2,1)	(0,0)
***Myotis emarginatus***	NT	(0,0)	(0,2)	(0,0)
***Myotis crypticus***	NE	(1,0)	(0,0)	(0,1)
***Nyctalus leisleri***	NT	(1,0)	(0,0)	(0,0)
***Pipistrellus kuhlii***	LC	(0,4)	(0,0)	(1,5)
***Pipistrellus pipistrellus***	LC	(0,1)	(0,0)	(0,0)
***Hypsugo savii***	LC	(0,4)	(0,0)	(13,76)
***Rhinolophus ferrumequinum***	VU	(0,0)	(1,0)	(0,0)
***Rhinolophus euryale***	VU	(0,0)	(3,7)	(0,0)

**Table 2:** Primers used for the detection of coronaviruses in the 240 bat (droppings or saliva) samples analysed. The amplification size is expressed as base pairs (bp). RdRp: RNA-dependent RNA polymerase.

Virus	Primer	Sequence	Amplification Size	Reference	Gene
**Coronavirus** **Coronavirus**	F	5′-GGGTTGGGACTATCCTAAGTGTGA-3′	251	[32]	RdRp
	R	5′-TAACACACAAACACCATCATCA-3′			
	F	5′-ACWCARHTVAAYYTNAARTAYGC-3′	452	[34]	RdRp
	R	5′-TCRCAYTTDGGRTARTCCCA-3′			

## Data Availability

Not applicable.
